# Pregnancy risk during menstrual cycle: misconceptions among urban men in India

**DOI:** 10.1186/s12978-017-0332-3

**Published:** 2017-06-12

**Authors:** Prashant Verma, Kaushalendra Kumar Singh, Anjali Singh

**Affiliations:** Department of Statistics, Banaras Hindu University, Varanasi, 221005 India

**Keywords:** Pregnancy risk, Menstrual cycle, Reproductive health, Discriminant analysis, Unwanted pregnancy, Abortion, Misconceptions, Fertile Window.

## Abstract

**Background:**

In India, where men take most decisions in the family, it is useful that they have adequate knowledge about pregnancy risks during women’s menstrual cycles. Since traditional contraceptive methods are still employed by a large population in India, the knowledge regarding the pregnancy risk during the menstrual cycle is indispensable. This research paper attempts to assess the knowledge among urban men in Uttar Pradesh, India about the fertile window of the menstrual cycle; it also attempts to discover the rationales behind the misconceptions about the concept.

**Methods:**

This study utilizes the baseline data of the Measurement, Learning, and Evaluation project for the Urban Reproductive Health Initiative in Uttar Pradesh. Descriptive Statistics has been used to assess the prevalence of knowledge among urban men regarding the concept. Using the Discriminant Analysis, we also investigate the rationales behind the misconceptions among urban men about the concept.

**Results:**

Only one-fifth of the men have the correct knowledge about the concept. Further, we find that education, societal perception, caste, and spousal discussion about the reproductive issues are the primary factors affecting the knowledge about the pregnancy risk during the menstrual cycle.

**Conclusions:**

There is an urgent need for sex education in the region to make the urban men more educated about the reproductive process of women; this may reduce unwanted births and abortion due to an unwanted pregnancy as well. The study promotes the higher education and motivates couples to discuss the reproductive health issues among them. In this manner, we can provide better reproductive health to the women of urban India.

## Plain English summary

In India, traditional contraceptive methods are employed by a large population, and most of the time decisions related to family planning are taken by the men of the household. Therefore, it is really necessary for men to have the correct idea of when a woman is most susceptible to pregnancy during the menstrual cycle. The study tries to assess the prevalence of such knowledge among urban men in Uttar Pradesh, India. Also, it attempts to investigate the rationals behind the misconceptions among men of the region under study. We have found that only one-fifth of the men have the correct knowledge about the concept. Further, education, societal perception, caste, and spousal discussion about the reproductive issues are found to be the most important components that affect the knowledge among men about the conception risk during the menstrual cycle. The study suggests promoting the sex education in urban Uttar Pradesh, especially in slum areas to educate the men regarding the reproductive functions of women. Also, the article promotes higher education and motivates couples to discuss the reproductive hurdles among them. These interventions can provide a better reproductive health to the women of urban Uttar Pradesh, India.

## Background

The effective contraceptive practice is usually measured by knowledge about the reproductive process and related issues. In India, where men take most family decisions, it is important that they have the adequate knowledge about the pregnancy risk during women’s menstrual cycle. In an American study, it is observed that only one-third of urban mothers interviewed twice, a year apart, answered correctly both times about when during the menstrual cycle a woman is most likely to become pregnant [1]. Recently, a research found that about 85% women do not have the correct knowledge of the fertile window of a menstrual cycle [2].

Menstruation is an important reproductive health function, yet it has been dealt with secrecy in India, [3]. Due to some cultural barriers, most couples rarely have a conversation regarding the menstruation and pregnancy risks during menstruation. A number of taboos and social and cultural restrictions still exists concerning menstruation, [4], [5], [6], and [7]. Researchers and policy makers often talk about woman’s knowledge regarding the procreative process [8], and [9], although men’s knowledge about the reproductive process is as indispensable as their decision making in the family. As it is argued, erroneous information about the risk of conception during the menstrual cycle may lead to increased fertility [10]. It is realized that only 18.4% of the men in rural eastern part of Uttar Pradesh had the correct information about the time of maximum conception risk during the menstrual cycle, while 43.2% did not have any idea about this concept [11].

Usually, the sperm can survive for three to five days in the fallopian tube and after ovulation; the released egg takes approx 12 to 24 hours to make its way through the fallopian tube. Thus, chances of pregnancy to occur are highest when a couple has intercourse without contraceptives one to two days before ovulation. A female usually ovulates 12–14 days after her periods. If a woman has a regular menstrual cycle length of 28 days, she will ovulate in the middle of the cycle, nearly 14 days after day one of her periods. In this connection, the 14th day of the cycle is the most susceptible day for fertilization of the egg [12]. If a woman has her menstrual cycle little longer, say 34 days, she will ovulate around 20 days after day one of her periods. In some cases, women know when they are ovulating by observing the changes in their body and the way they feel. Some quintessential measures are breast tenderness, hefty and denser vaginal discharge, tightness in the abdomen; however, these body changes are difficult to be understood by couples. Many others do not have any noticeable symptoms.

In India, the traditional contraceptive methods are still employed by a large population; therefore, the misinformation about the fertile window during MC may lead to the failure of the traditional contraception methods. The knowledge about the conception risk during the menstrual cycle is essential to ward off the unwanted births and abortions due to unwanted pregnancies. Against this background, this study estimates the prevalence of knowledge about the conception risk during menstrual cycle among urban men of Uttar Pradesh, India. In this connection, men have been asked when during the menstrual cycle they think women are most susceptible to the risk of conception and we attempt to discover the rationales behind the misconceptions among urban men about the concept.

## Methods

For this study, we use the baseline data of the Measurement, Learning, and Evaluation (MLE) Project for the Urban Reproductive Health Initiative (URHI) in Uttar Pradesh, India. URHI is a multi-national study involving, Kenya, Nigeria, and Senegal that assess contraceptive behavior, awareness, and quality among poor belonging to urban areas. The Carolina Population Centre at the University of North Carolina at Chapel Hill commenced the Project, in cooperation with the International Centre for Research on Women (ICRW) sponsored by the Bill & Melinda Gates Foundation to undertake an evaluation of the URHI programs in Uttar Pradesh. The baseline data were collected in four cities, Agra, Aligarh, Allahabad and Gorakhpur. A total of 6431 currently married men aged 18–54 were interviewed in these towns. The comprehensive survey response rate was 88%. A two-stage sampling approach was employed to collect the sample for each city. Cities were split into slum and non-slum as primary sampling units based on ground truthing and satellite imagery. Questions about awareness of contraceptive methods, fertility desires, attitudes toward reproductive health, contraceptive use by themselves or their wives, the pregnancy risk during the menstrual cycle, were asked to the men belonging to urban Uttar Pradesh, India.

As Table 2 shows, the dependent variable in this study refers to the response of men regarding the time of highest risk of conception during the menstrual cycle of women. There are five choices of outcome: “just before the menstrual cycle begins”, “during the cycle”, “right after the period ends”, “halfway between the two periods” and “do not know”. For the purpose of analysis, the dependent variable was re-coded into a new variable which has only two categories: (1) men who have the false information or do not know about the concept of pregnancy risk during menstrual cycle, and (2) men who have the correct information about the time at which the conception risk is highest i.e. (halfway between the two periods) approximately on 14th day of usual menstrual cycle of 28 days.

**Table 1:** Distribution of the variables

Variables	Number of cases	Percentage distribution	Variables	Number of cases	Percentage distribution
Ever Discussed FP with Wife			Residence		
Yes	4193	80.62	Non-Slum	2675	51.43
No	1008	19.38	Slum	2526	48.57
Caste			Religion		
SC/ST	1158	22.26	Hindu	4123	79.27
OBC	2031	39.06	Non Hindu	1078	20.73
General	2012	38.68			
Wealth Status			Society Encourages MFP Methods		
Lower	826	15.88	Yes	3029	58.24
Middle	3304	63.53	No	580	11.15
Upper	1071	20.59	Don’t know	1592	30.61
Media Exposure			Information about Pregnancy Risk		
Exposed to media	4519	86.89	True Information (Know the concept)	1051	20.21
Not exposed to media	682	13.11	False Information (Don’t Know)	4150	79.79
Age			Education		
Below 30 Yrs	1212	23.30	No Education	518	09.96
(30–34) Yrs	924	17.77	Primary	676	13.00
(35–39) Yrs	993	19.09	Secondary	2811	54.04
(40–44) Yrs	884	17.00	Higher	1196	23.00
Above 44 Yrs	1188	22.84			

### Discriminant analysis

“Discriminant analysis (D.A.) is a statistical technique which allows us to study the differences between two or more groups of objects concerning several variables simultaneously.” [13]. Discriminant analysis does the same analysis as linear regressions, by predicting an outcome; however, in multiple linear regression, the dependent variable is an interval variable so that the combination of explanatory variables will provide estimated mean population Y values for given values of the weighted sum of X values (Predictor) through the regression function. Discriminant analysis is used when the dependent is a categorical variable with the predictors of interval level, such as years of education, income, and age; although one can use dummy variables as predictors similar to multiple regression.

### Discriminant analysis, linear equation

The form of the discriminant analysis equation or function is:

$$ \mathrm{D}={\mathrm{V}}_{\mathbf{1}}{\mathrm{X}}_{\mathbf{1}}+{\mathrm{V}}_{\mathbf{2}}{\mathrm{X}}_{\mathbf{2}}+{\mathrm{V}}_{\mathbf{3}}{\mathrm{X}}_{\mathbf{3}}+\dots \dots \dots .+{\mathrm{V}}_{\mathrm{i}}{\mathrm{X}}_{\mathrm{i}}+\mathrm{a} $$

where;

D = Discriminant function or discriminant score

V = The discriminant function coefficient or weight for that variable

X = Respondent’s score for the particular predictor variable

a = A constant

i = the number of predictor variables

This equation is similar to a regression equation. The V_i_’s are the discriminant coefficients analogous to the b_i_’s in the regression equation. Good predictors contain larger weights in discriminant function. The equation should hold strong discriminatory power between groups since the discriminant function is supposed to maximize the distance between the categories of study variable. Thus, the D.A. also explores differences between groups on the basis of different characteristics (Regressors) of the cases, indicating which components (Regressors) contribute most to the group separation.

The number of discriminant functions is one less than the number of groups or category. There is only one function for the D.A. of this problem since our dependent variable has only two classes. In our problem the dependent variable, knowledge about the pregnancy risk during menstrual cycle has been classified into two categories; one has the accurate knowledge about the fertile window during the menstrual cycle, and the other category does not have the actual idea of the concept. Since the predictors, involved in our D.A, are not at interval level, we have created dummy variables for each category of predictor variables. In this study, the D.A has been performed for slum area and non-slum area separately.

**Fig. 1:**
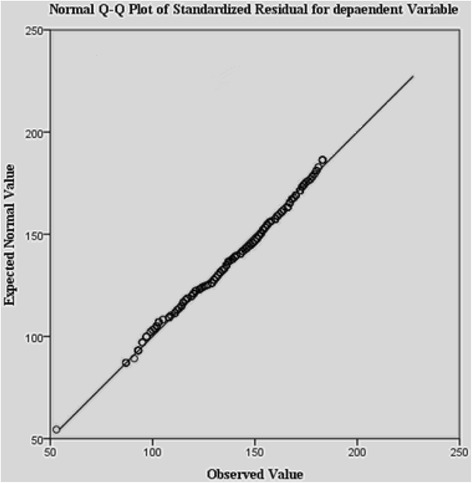
Normality check for the data

The paramount assumptions required to be tested to check the compatibility of data with D.A are normality and homoscedasticity. We have used Levene’s test of equality of error variances to verify the homogeneity of variance (homoscedasticity). As a result of the Levene’s test, the null hypothesis that the error variance of the dependent variable is equal across groups has been accepted (*p* < .05). Therefore, it can be concluded that the data hold the homoscedasticity assumption. Further, the normal Q-Q curve for the standardized residuals has been plotted to check the normality assumption. After having a glance at Fig. 1, it is observed that the residuals are normal in nature. Since the data fulfill the assumptions of homoscedasticity and normality, the discriminant analysis has been applied for analysis. SPSS software has been used for the above analysis.

## Results

Table 1 reveals the percentage distribution of the variables included in the analysis. We find that about 80% of the men had a discussion about family planning with their wives at least once, while slightly less than one-fifth never had any discussion. It is essential to note that only one-fifth of the men have the correct information about pregnancy risk during the menstrual cycle, while the remaining four-fifth of them do not have the accurate information about this concept.

Table 2 gives the percentage distribution of men regarding their perception or knowledge regarding the time duration, which has the highest pregnancy risk during the menstrual cycle. We find that in the non-slum areas about 21% of the men respond that the maximum risk of gestation occurs halfway between two periods that is the accurate information regarding the concept, while in slum areas about 19% men have the actual information. Table exhibits that about 13% among uneducated men, about 18% among primarily educated men, about 19% among secondary educated men and 24% among highly educated men have the accurate information about the concept. It is observed that 24% among the men who discussed family planning with their wives and only about 5% among the men who did not discuss family planning with their wives possess the correct idea about the pregnancy risk during MC.

**Table 2:** Percentage distribution of men regarding the perception about pregnancy risk during menstrual cycle

Highest chance of conception		Just before period begins	During period	Right afterperiod ends	Halfway betweentwo periods	Don’t know	Total count
Residence	Non-Slum	1.16	06.95	59.07	21.12	11.70	2675
	Slum	1.39	10.33	55.42	19.24	13.62	2526
Caste	SC/ST	1.21	10.71	57.43	15.63	15.02	1158
	OBC	1.58	08.71	56.97	21.27	11.47	2031
	General	0.99	07.26	57.55	21.77	12.43	2012
Religion	Hindu	1.33	08.68	60.44	17.12	12.43	4123
	Non Hindu	1.05	08.41	53.77	22.06	15.24	1078
Education	No Education	2.80	08.41	56.07	13.08	19.64	518
	Primary	0.84	11.81	53.43	17.99	15.93	676
	Secondary	1.37	09.33	56.81	19.09	13.40	2811
	Higher	1.18	05.27	60.51	24.40	08.64	1196
Age	Below 30 Yrs	0.99	09.57	57.84	17.24	14.36	1212
	(30–34) Yrs	1.30	07.14	61.36	19.59	10.61	924
	(35–39) Yrs	1.21	09.06	56.80	20.44	12.49	993
	(40–44) Yrs	1.58	07.58	56.45	21.61	12.78	884
	Above 44 Yrs	1.35	09.09	54.63	22.47	12.46	1188
Media Exposure	Exposed to media	1.33	08.92	57.47	20.40	11.88	4519
	Not exposed to media	0.88	06.45	56.16	18.91	17.60	682
Society encourages MFP methods	Yes	1.16	08.02	53.55	24.30	12.97	3029
	No	3.10	15.17	57.59	13.10	11.04	580
	Don’t know	0.82	07.29	64.32	15.01	12.56	1592
Wealth Status	Lower	1.21	11.50	54.96	17.19	15.14	826
	Middle	1.30	07.63	58.23	20.25	12.59	3304
	Upper	1.21	09.34	56.21	22.41	10.83	1071
Discussed FP with wife	Yes	1.14	07.35	56.79	23.83	10.89	4193
	No	1.79	13.79	59.42	05.16	19.84	1008

After having a glance at the table, it can be concluded that the highest percentage men have given the response that the maximum risk of conception occurs right after the menstruation period ends, and its percentage is more than 50 for each category of variables under consideration. Further, it can also be seen from the table that almost one-fifth respondents, do not have any idea about the concept among the illiterate men and men who have never discussed family planning with their wives.

Table 3 presents the test of equality of group means for the different variables taken into consideration for the slum area. In the table, group 1 includes the respondents who do not have the correct idea about the concept and group 2 includes the respondents who have the accurate information about the notion of pregnancy risk during MC. Since all the exogenous variables are made binary (In form of 0 or 1), the mean provides the proportion of respondents in a particular category of the variable. The table depicts that the men belonging to Hindu religion have a higher proportion (81%) in group 1, and the men belonging to the non-Hindu religion have a greater proportion in group 2 (41%). It shows that men belonging to other religions have better information compared to the men belonging to the Hindu religion. Further, it is found that uneducated men have a higher proportion (10%) in group 1 compared to group 2 (7%) and the men having higher education have a greater share in group 2 (20%). The above finding indicates that men, having higher education keep better information about the concept compared to the men with no education.

**Table 3:** Test of equality of group means for slum

	Predictors	Mean	S.D	Mean	S.D	*p* Value
		(Group 1)	(Group 1)	(Group 2)	(Group 2)	
Caste	SC / ST	0.32	0.47	0.22	0.41	0.01
	OBC	0.42	0.49	0.44	0.49	0.43
	General	0.26	0.44	0.34	0.47	0.01
Religion	Hindu	0.81	0.38	0.59	0.49	0.01
	Non Hindu	0.19	0.38	0.41	0.48	0.01
Education	No Education	0.10	0.17	0.07	0.11	0.02
	Primary Education	0.12	0.39	0.11	0.37	0.32
	Secondary Education	0.64	0.49	0.62	0.48	0.98
	Higher Education	0.14	0.37	0.20	0.40	0.04
Age	Age Below 30 Yrs	0.26	0.44	0.22	0.41	0.06
	Age 30 to 34 Yrs	0.18	0.39	0.19	0.39	0.65
	Age 35 to 39 Yrs	0.19	0.40	0.19	0.39	0.75
	Age 40 to 44 Yrs	0.16	0.37	0.18	0.38	0.23
	Age Above 44 Yrs	0.20	0.40	0.22	0.41	0.43
Media Exposure	Exposed to Media	0.84	0.35	0.85	0.31	0.45
	Not Exposed to Media	0.16	0.37	0.15	0.35	0.45
Society Encourages MFPM	Society Encourages MFP Method	0.53	0.49	0.70	0.45	0.00
	Doesn’t Encourage MFP Method	0.13	0.33	0.07	0.25	0.00
	Don’t Know about society	0.34	0.47	0.23	0.42	0.00
Wealth Status	Lower Wealth	0.22	0.41	0.17	0.38	0.04
	Middle Wealth	0.65	0.47	0.67	0.47	0.48
	Upper Wealth	0.13	0.33	0.15	0.36	0.13
Discussed FP	Discussed FP with Wife	0.75	0.41	0.94	0.27	0.00
	Don’t Discuss FP with Wife	0.25	0.43	0.06	0.24	0.00

Group 1 includes the respondents who do not have the correct knowledge and group 2 includes the respondents who have the accurate information about the fertile window of the menstrual cycle

The table shows that the media exposure is not a significant factor for the group separation regarding the knowledge about the conception risk during the MC; this might be due to a high correlation between the media exposure and the educational attainment of men in urban Uttar Pradesh. The finding says that the men who dwell in a society that supports MFPM have more actual information compared to the men living in a society that does not support MFPM and the men who do not know about the view of society regarding the MFPM.

Further, it is found that the men who discuss family planning with their wives have a higher proportion (94%) in group 2 and the men who do not discuss the family planning have a higher percentage (25%) in group 1. This finding leads to the statement that the men who discuss family planning with their wives have more actual information regarding the concept of highest pregnancy time during MC compared to the men who do not talk about the family planning with their partner.

Table 4 presents the test of equality of group means for the different variables taken into consideration for the non-slum area. It can be regarded from the table that the variables like the perception of society about MFPM, religion, and discussion of the respondent with the wife about family planning reflects the same results as we have found in the analysis for slum area. Further for non-slum population, caste has not been noticed as a significant factor for group separation. The table reflects that for non-slum population, wealth status of men is not an important variable; this may be due to that other social and cultural factors are more dominant; also wealth status is highly associated with educational attainment.

**Table 4:** Test of equality of group means for non slum

	Predictors	Mean	S.D	Mean	S.D	*p* Value
		(Group 1)	(Group 1)	(Group 2)	(Group 2)	
Caste	SC / ST	0.15	0.35	0.13	0.34	0.26
	OBC	0.35	0.47	0.39	0.48	0.13
	General	0.50	0.50	0.48	0.50	0.50
Religion	Hindu	0.83	0.37	0.73	0.44	0.01
	Non Hindu	0.17	0.36	0.27	0.47	0.01
Education	No Education	0.08	0.11	0.07	0.11	0.99
	Primary Education	0.10	0.30	0.09	0.28	0.31
	Secondary Education	0.51	0.49	0.42	0.50	0.01
	Higher Education	0.31	0.47	0.42	0.49	0.01
Age	Age Below 30 Yrs	0.22	0.41	0.18	0.38	0.03
	Age 30 to 34 Yrs	0.18	0.38	0.16	0.36	0.26
	Age 35 to 39 Yrs	0.19	0.39	0.20	0.39	0.55
	Age 40 to 44 Yrs	0.17	0.37	0.18	0.38	0.68
	Age Above 44 Yrs	0.24	0.42	0.28	0.45	0.03
Media Exposure	Exposed to Media	0.90	0.32	0.90	0.29	0.77
	Not Exposed to Media	0.10	0.30	0.10	0.28	0.77
Society Encourages MFPM	Society Encourages MFP Method	0.57	0.49	0.70	0.45	0.00
	Doesn’t Encourage MFP Method	0.11	0.31	0.07	0.26	0.00
	Don’t Know about society	0.32	0.46	0.23	0.41	0.00
Wealth Status	Lower Wealth	0.11	0.31	0.10	0.30	0.39
	Middle Wealth	0.62	0.48	0.61	0.48	0.68
	Upper Wealth	0.27	0.44	0.29	0.45	0.29
Discussed FP	Discussed FP with Wife	0.79	0.40	0.96	0.19	0.00
	Don’t Discuss FP with Wife	0.21	0.43	0.04	0.22	0.00

Group 1 includes the respondents who do not have the correct knowledge and group 2 includes the respondents who have the accurate information about the fertile window of the menstrual cycle

Tables 5, and 6 provide the significance testing of the Discriminant functions for slum and non slum data respectively. Tables provide information on each of the discriminant functions (equations) produced. The maximum number of discriminant functions produced is the number of groups minus 1. In this study only two groups are considered, thus only one function is displayed. The canonical correlation is the multiple correlation between the predictors and the discriminant function. With only one function, it provides an index of overall model fit which is interpreted as being the proportion of variance explained (R^2^). In Table 5 a canonical correlation of 0.926 suggests that the model explains 85.75% of the variation in the grouping variable for slum data; while as per the Table 6, a canonical correlation of 0.901 exhibits that the model explains 81.18% of the variation for non slum data. Wilks’ Lambda is the ratio of within-groups sums of squares to the total sums of squares. This is the proportion of the total variance in the discriminant scores not explained by differences among groups. This is a measure of how well each function separates cases into groups. Smaller values of Wilks’ lambda indicate the greater discriminatory ability of the function. The Tables 5, and 6 indicate highly significant discriminant function (*p* < 0.01) with the values of Wilks’ Lambda, i.e. 0.328 and 0.388 for slum and non slum analysis respectively. Thus, our discriminant function is statistically significant for group separation.

**Table 5:** Testing of discriminant function for slum

Test of function(s)	Canonical correlation	Eigenvalue	Wilks’ Lambda	df	Sig.
1	0.926	0.822	0.328	16	0.00

**Table 6:** Testing of discriminant function for non slum

Test of function(s)	Canonical correlation	Eigenvalue	Wilks’ Lambda	df	Sig.
1	0.901	0.813	0.388	16	0.00

## Discussion

This study shows that relatively small proportion of urban men has correct information about the fertile window of women’s menstrual cycle. Considering that almost four-fifth of married men in both slum and non-slum areas of urban Uttar Pradesh do not have the correct knowledge of the concept, there is an urgent need for sex education. Classical contraceptive methods are also used by a huge number of couples in the region. Therefore, the scientific knowledge of women’s reproductive system will help reduce the number of abortions resulting from unwanted pregnancies and unwanted births, which may be a cause of high fertility in Uttar Pradesh, India.

The study depicts that the higher education results into a better knowledge about the conception risk during the menstrual cycle. Literacy and employment can bring the wealth condition up; therefore literacy is the only way to get rid of such misinformations regarding the reproductive biology of women, which is why study suggests promoting the higher education. Our perception about something is built according to the society; in which we are living. Due to this, it is found that men living in a society, that encourages the MFPM, have better information about the fertile window than who are residing in a society that does not support MFPM. Thus, to educate people, it is necessary to educate the society as a whole. Also, this study suggests urban men, to have interaction with the society and discuss the reproductive health and other related issues like family planning, etc.

The effective inter-spouse communication on matters, related to family planning is very crucial for the success of family planning programs [14], and [15]. In India due to various cultural barriers and customs, even husband and wife feel shy to discuss the sensitive issues like family planning and reproductive process. Sharing the accurate knowledge with the spouse regarding the fertile window during MC will reduce the misconceptions among the urban men in the region. The study recommends couples to talk about the sexual health and other relative issues with their spouses so that they can be aware of the different technicalities related to reproductive health and family planning.

The main limitation of the study is that it considers only one state (Uttar Pradesh) of India, though there are several states which are bearing the same challenges. There exists a huge diversity among the Indian states in terms of education, socio-economic conditions, culture, and norms. Thus, one can not generalize the findings for other states of India. Also, the study involves men only from the urban residence, though the men dwelling in rural settings are less educated and might have less knowledge regarding the fertile window of MC.

## Conclusions

The study has its huge significance for reducing the unwanted pregnancies and unwanted births due to the misinformation among men regarding the fertile window of the menstrual cycle. Unwanted conceptions may cause excess abortions, which is harmful to the reproductive health of the women and at the other side unwanted births also cause the high fertility. Thus, the accurate information about the fertile window is essential to eliminate the above two factors. Educating the urban men about the fertile window and other reproductive functions is the ultimate solution to remove the misconceptions regarding the concept among them. The discussion between couples regarding the reproductive biology of women will help to increase the knowledge among the men of urban Uttar Pradesh about the fertile window of the menstrual cycle.

## Abbreviations

DA: Discriminant analysis
MC: Menstrual cycle
MFPM: Modern family planning methods
MLE: Measurement, learning, and evaluation
OBC: Other backward castes
SC/ST: Scheduled caste/scheduled tribes

## References

[1] Presser HB (1977). Guessing and misinformation about pregnancy risk among urban mothers. Fam Plan Perspect.

[2] Begum S, Dwivedi SN, Mittal S, Pandey A (2013). Knowledge and practice of periodic abstinence among women in India. Open Journal of Preventive Medicine.

[3] Kumar A, Srivastava K (2011). Cultural and social practices regarding menstruation among adolescent girls. Social Work in Public Health.

[4] Dhingra R, Kumar A, Kour M (2009). Knowledge and practices related to menstruation among tribal (gujjar) adolescent girls. Ethno-Med.

[5] Paul D (2007). Knowledge and practices of adolescent girls regarding reproductive health with special emphasis on hygiene during menstruation.

[6] Singh AJ (2006). Place of menstruation in the reproductive lives of women of rural North India. Indian J Community Med.

[7] Mahon T, Fernandis M (2010). Menstrual hygiene in South Asia: a neglected issue for WASH (water, sanitation and hygiene) Programmes. Gend Dev.

[8] Baridalyne N, Reddaiah VP (2004). Menstruation: knowledge, beliefs and practices of women in the reproductive group residing in an urban resettlement colony of Delhi. Health and Population Perspectives.

[9] Deo DS, Ghattargi CH (2005). Perceptions and practices regarding menstruation a comparative study in urban and rural adolescent girls. Indian J Community Med.

[10] Mendelbaum DG (1974). Human fertility in India.

[11] Yadava RC, Mishra CP (2012). Poverty, under-nutrition and fertility nexus in rural eastern Uttar Pradesh.

[12] Wilcox J (2000). The timing of the “fertile window” in the menstrual cycle: day specific estimates from a prospective study. BMJ.

[13] Klecka WR. “Discriminant analysis” (Book Volume No. 19). ISBN: 9780803914919, Sage publishing, 1980. https://us.sagepub.com/en-us/nam/discriminantanalysis/book440.

[14] Bogue D, Kiser CV (1962). Some tentative recommendations for a ‘sociologically correct’ family planning communication and motivation programme in India. Research in family planning.

[15] Acharya R, Sureender S (1996). Inter-spouse communication, contraceptive use and family size: relationship examined in Bihar and Tamil Nadu. J Fam Welf.

